# What is the intermediate host species of SARS-CoV-2?

**DOI:** 10.2217/fvl-2020-0390

**Published:** 2021-02-04

**Authors:** Thomas Friend, Justin Stebbing

**Affiliations:** ^1^Department of Surgery and Cancer, Imperial College, London, UK

**Keywords:** ACE2, bat, COVID-19, mutations, pangolin, SARS-COV-2

## Abstract

Dissecting the published evidence on the intermediate host species of SARS-CoV-2. An editorial review of the proximal origins of SARS-CoV-2, what may have been missed and why it matters.

At the genome-wide level, SARS-CoV-2 is similar to the coronavirus’s two known predecessors found in circulation in humans to date: SARS-CoV and MERS-CoV (79.5 and 50% sequence identity respectively) [1]. The SARS-CoV-2 spike protein, however, exhibits 10–20-times higher affinity for target cell angiotensin-converting enzyme 2 (ACE2) receptors in humans [1]. The importance of these differences arises from SARS-CoV-2’s infection pathway.

Several viruses in circulation in animals with a greater degree of homology to SARS-CoV-2 have been identified and proposed to be likely progenitors of SARS-CoV-2. RaTG13, identified in 2016 and the most likely contender identified thus far, is a bat coronavirus which shares 96% of its genome with SARS-CoV-2 [2,3]. Two primary factors, however, have led the scientific community to conclude that humans were initially infected by a virus that circulates in a second intermediate host species.

First, estimated mutation rates deduced from observations in humans suggests RaTG13 shared a common ancestor with SARS-CoV-2 between 25 and 65 years ago [2]. This timeline is incongruent with SARS-CoV-2’s emergence approximately 1 year ago and low genetic diversity observed across the initial viral sequences obtained from humans [2].

Second, there are differences in the genetic sequences encoding the spike protein, which the virus relies on to enter human cells and accounts for many of SARS-CoV-2’s unique pathophysiologic properties, including increased infectivity versus RaTG13 [1,2]. It is widely accepted that a bat with RaTG13 infected another species and that animal subsequently infected humans with SARS-CoV-2, as we know it today. Researchers have, therefore, continued searching for the progenitor species that infected humans: a coronavirus with similar binding affinities for human ACE2 and a viral genome that shows a greater degree of similarity to SARS-CoV-2 [3]. A coronavirus in pangolins, for example, was found to have a receptor-binding domain in its S1 spike that was almost an identical match to that of SARS-CoV-2 but with greater differences at the genome-wide level, ruling it out as the progenitor of SARS-CoV-2 [2,3].

Mapping the genomic differences, or phylogenies, of viruses typically allows epidemiologists to infer the most common recent ancestor (MRCA) for a set of viral sequences and can be an invaluable tool in tackling pandemics [4]. For example, in April 2009, genetic analysis of 11 sequences of H1N1 influenza indicated that the MRCA existed at or before 12 January 2009, enabling epidemiologists to infer the origins of the virus as well as make early estimates of the basic reproduction number (R0) [4]. The current pandemic has elicited similar approaches; mass sequencing initiatives such as GISAID (www.gisaid.org) have enabled scientists to establish the rate of evolution measured in real units of time and map the phylogenies of the SARS-CoV-2 through platforms such as Nextstrain (https://nextstrain.org/) [2,3]. Despite this unprecedented allocation of resources, efforts to confirm the mutation rate of SARS-CoV-2 have seemingly belied identifying the origins of SARS-CoV-2, and questions critical to our understanding of the origins of SARS-CoV-2 remain unanswered [2,3,5].

Uncertainty as to origins of viral outbreaks is not a new phenomenon: the identification of RaTG13 came as the result of a five-year surveillance program of bats from a single cave located in the Yunnan Province of China as part of an effort to identify the origins of the SARS pandemic [6–8]. The research findings, published in 2017 in *PLoS Pathogens*, identified 11 new coronaviruses, one of which was hypothesized to be the direct progenitor of the SARS outbreak [8]. Frequent recombination events and, significantly, bat co-infection with multiple SARS-related CoVs (SARS-CoVs) were also detailed in the research [8]. The same team that led this ground-breaking surveillance initiative also published an earlier paper that identified four novel SARS-CoVs, three of which were capable of efficient reproduction in human airways [8]. Compounding the significance of this research was the finding of co-infections across all six bat species represented. Given that all 138 infected bat specimens were sampled from the same habitat, this research highlights the requirement to assess the spatio-temporal effects and population dynamics in the ecological compartments of virus host species when estimating historic mutation rates and their proximal origins.

Most critical to our inferences in phylogenetic modeling in the current pandemic, however, is the documented reliance on transgenic animal models expressing human ACE2 as reported by the bat surveillance programs [8]. This was also proposed in a letter from the lead scientist to *Science* magazine in 2020 [9]. The use of chimeras is significant given that Bayesian phylogenetic and phylodynamic data integration would require the common ancestor of the entire virus population to have existed earlier than the MRCA of the virus sample in order for timing estimates to be accurately made [4]. If an animal model or a descendant of one were to be the progenitor species of SARS-CoV-2, this principle would not hold, and established estimates as to the timing of the MRCA would be overestimated. This point is underscored by the fact that RNA viruses with narrow host ranges, including SARS-CoV-2 and RaTG13, evolve quickly after blind serial passage *in vitro* and *in vivo*, a process relied upon to promote adaptation to new host species and also required for the development of animal models [10–14].

If we are to re-evaluate our assumptions in modeling the evolutionary history of SARS-CoV-2, we must also consider single nucleotide variants (SNVs) and their impact on presumed rates of evolution. SNVs are acquired in RNA viruses, including coronaviruses, at a rate 103–107-times greater per nucleotide copied than DNA viruses [10,11]. During replication SNVs are purged or retained resulting in the formation of quasi-species with greater fitness than that of the parent or ‘master’ sequence [11]. This increase in fitness is accounted for by the acquisition of mutations across SNVs, which result in selection, competition and genetic drift acting upon the entire viral ‘swarm’ [11]. Frequently present in recombinant coronaviruses, such as SARS-CoV-2 and RaTG13, quasi-species allow for either the evolution of the ‘master’ consensus sequence or the entirety of its mutant spectrum [10,11,15]. The optional duality of these simultaneous processes and corresponding outcomes allows for either stasis or rapid evolution in the observed consensus sequence (10^-^^4^ substitutions per site per year versus 10^-^^1^–10^-^^2^ substitutions per site per year) [10,11]. It follows, therefore, that the calculation of the nonsynonymous to synonymous (dN/dS) substitution rate ratio of RNA in the phylogenetic analysis should account for these factors, where appropriate [15]. Moreover, SNVs serve as a reminder that observed stasis does not necessarily equal a mutation rate lower than the RNA genome replication rate, nor does rapid evolution imply higher-than-average mutation rates [10,11]. Furthermore, changes to the genetic composition of viral isolates in cell cultures, even when adaption periods are short, highlight that consensus sequences should not be interpreted as a proxy for fitness [10,11,15].

The interplay of the phenomena discussed thus far, namely the effect of environmental and host factors in combination with the cellular and subcellular processes taking place dependent on the virus being studied, are best illustrated in MA15, a mouse-adapted strain of SARS-CoV. On initial infection with SARS-CoV-1, wild-type mice were found not to express the same SARS-CoV infection phenotypes seen in humans [12–14]. Following 15 passages of SARS-CoV in the respiratory tract of young BALB/c mice, the mice developed disease phenotypes similar to humans [12]. The development of the MA15 mouse model, therefore, demonstrates both the results of immune senescence and the amplification of pathogen phenotype in the process of developing relevant animal models. It is also worth noting that research on other RNA viruses, including foot-and-mouth disease, shows cellular tropism when serial passthrough is conducted and so the expectation of a more specialized infection of the cellular type to which the virus has been adapted cannot be relied upon [12,16].

Beyond considering animal models in our evaluation of the proximal origins of SARS-CoV-2, consideration of the contextual factors discussed thus far are also critical to predict the outcome of and respond to the current pandemic. The accelerated evolution of SARS-CoV-2 in a 45-year old patient with a compromised immune system as a result of severe antiphospholipid syndrome documented in correspondence in the *NEJM* published December 2020 highlights this point. Through viral load assays and phylogenetic analysis researchers were able to conclusively provide evidence of accelerated evolution of the virus, ruling out a secondary infection [17]. The parallels between the environment in which SARS-CoV-2 evolved in this patient and knockout mice used to develop chimeras, specifically serial passage and immune senescence and subsequent disease phenotype amplification, warrants further research into the exact mechanisms involved and their occurrence in both humans and animal models. Intensification of genomic surveillance where a cluster of infections in patients with immune disorders, for example, treatment centers for those with immune disorders, is therefore warranted until concrete findings are made.

In summary, to fully understand the origins of SARS-CoV-2 we must adjust our operating assumptions. First and foremost, the scope of hosts must include those where serial passage has taken place or is likely to occur, even if they are not naturally occurring as is the case of knockout mice with human ACE2 receptors. Second, we must explore the effect of both host types on the corresponding processes occurring at cellular and subcellular levels in viruses and environmental factors including population size and fitness of the animal hosts themselves.
